# Supplementary material for: Essential criteria for reporting of aromatherapy-focused research in humans: An international Delphi consensus study protocol
Source: PLoS One. 2025 Mar 24;20(3):e0318379. doi: 10.1371/journal.pone.0318379 (PMC11932481; doi:10.1371/journal.pone.0318379)
Supplement: S1 File — (DOCX) [file pone.0318379.s001.docx]

**DELPHI CONSENSUS STUDY**

**Principal Investigator: Marian Reven, West Virginia University, United States, and the Aromatic Research Quality Appraisal Taskforce (ARQAT)**

**Participant Information Letter:**

**Invitation to join this international study.** The Link to begin participation is in the Cover Letter. Clicking the link will indicate your agreement to participate in this study. Please read this information sheet carefully and contact the Principal Investigator, Dr. Marian Reven, at [marian.reven@hsc.wvu.edu](mailto:marian.reven@hsc.wvu.edu) with any questions.

**Title: Essential Criteria for Reporting of Aromatherapy-Focused Research in Humans: An International Delphi Consensus Study**

**1. What is the study about?**

The study is designed to determine what items are considered necessary to include in aromatherapy-focused research.

The rise in aromatherapy-focused research parallels the increased global interest in aromatherapy using essential oils for support with illness, wellness, and quality of life. Promising evidence corroborates the role of aromatherapy in enhancing symptom management and health-related quality of life in various health areas.

The objective is to conduct an international [Delphi](https://www.rand.org/pubs/commentary/2023/10/generating-evidence-using-the-delphi-method.html) consensus study, aimed at selecting a core set of items to be considered in aromatherapy-focused research. These items will be an extension to existing reporting guidelines for various study designs including randomized controlled trials, observational studies, case reports, and other studies as applicable.

You have been invited to participate because of your expertise as a certified aromatherapy professional, researcher, and/or academic involved in aromatherapy-focused research.

This Participant Information Statement provides you with information about the research study. Knowing what is involved will help you decide if you want to take part in the research. Please read this sheet carefully and ask questions about anything that you don’t understand or want to know more about.

Participation in this research study is voluntary. It is up to you whether you wish to take part or not. By giving your consent to take part in this study you are telling us that you:

- Understand what you have read.
- Agree to take part in the research study as outlined below.
- Agree to the use of your personal information as described.

**2. Who is running the study?**

This project is administered through West Virginia University.

The Principal Investigator (PI) is Dr. Marian Reven

The Board of Directors of the Aromatic Research Quality Appraisal Taskforce ([ARQAT)](https://www.arqat.org/) is:

| Name | Affiliation | email |
| --- | --- | --- |
| Dr. Marian Reven, PI | West Virginia University | Marian.reven@hsc.wvu.edu |
| Dr. Kelly Ablard | Airmid Institute | Kelly.ablard@gmail.com |
| Dr. Esther Joy Bowles | University of New England | ejoybowles@gmail.com |
| Michelle Cohen | University of Maryland Medical Center | Justbe@rxcare4u.com |
| Denise Joswiak | Essential Health | joswiakdenise@gmail.com |
| Amanda May-Fitzgerald | Wild at Heart Botanicals | amandamayfitzgerald@gmail.com |
| Dr. Marilyn Peppers-Citizen | Maryland University of Integrative Health | youdowellness@gmail.com |
| Dr. Jerelyn Resnick | University of Washington | jerelyn@uw.edu |
| Bethany Unger | Veritas Aromatics | Dscents4you@gmail.com |

**3. Funding for this project**

This project is funded by several entities including the Gattefossé Foundation for open access publication of the results and by the West Virginia University Ruth and Robert Kuhn Nursing Research Fund for the in-person meeting in September 2024.

If you agree to participate, you will take part in a Delphi survey. The Delphi method is a form of survey where experts answer questionnaires in two or more rounds. After each round, the experts are provided with an anonymized summary of the previous round of responses. Then experts are encouraged to revise their earlier answers in light of the replies from other members of the group. **In this study, we will anonymize and summarize the results from up to four rounds of survey with the aim of reaching a consensus.**

**4. How much time will be involved?**

Each Delphi survey round could take between 1 to 3 hours. The first round is usually the most time intensive.

Study data are collected and managed using REDCap electronic data capture tools hosted at West Virginia University (Harris et al., 2009, 2019). REDCap (Research Electronic Data Capture) is a secure, web-based software platform designed to support data capture for research studies, providing 1) an intuitive interface for validated data capture; 2) audit trials for tracking data manipulation and export procedures; 3) automated export procedures for seamless data downloads to common statistical packages; and 4) procedures for data integration and interoperability with external sources.

**5. Who can take part in this study?**

We will accept those who wish to participate and who have qualifications as aromatherapy professionals, researchers, and/or academics involved in aromatherapy-focused research. Eligible researchers, academics who are proficient in reading and writing English, include those with an active or past role in aromatherapy-focused research, and those in the aromatherapy community involved in recognized education - all of whom have an interest in research quality, and reporting standards and guidelines.

Aromatherapy and health care professionals, who are proficient in reading and writing English, include aromatherapists with at least 400 hours of training from a recognized aromatherapy program. This also encompasses aromatherapists with backgrounds in pharmacy, chemistry, and nursing, as well as health care professionals with at least 200 hours of recognized aromatherapy education.

Our goal is to have a large and diverse group representing a wide global, research, practice, and academic perspective.

**6. Do I have to participate in the study? Can I withdraw from the study once I've started?**

Participating in this study is completely voluntary and you do not have to take part. Your decision whether to participate or not will not affect your current or future relationship with the researchers.

If you decide to take part in the study and then change your mind later, you are free to withdraw. Whatever your decision, please be assured that it will have no consequences for you. Withdrawal from the study can be organized by contacting the Principal Investigator, Dr. Marian Reven at [marian.reven@hsc.wvu.edu](mailto:marian.reven@hsc.wvu.edu) or phone +1 304-293-3399.

**7. How will my personal information be protected?**

After each round, participants in the next round are provided with an anonymized summary of the previous round of responses. Then participants are encouraged to review and revise their earlier answers in light of the replies and input from other members of the group. In this study, we will anonymize and summarize the results from up to four rounds of survey with the aim of reaching a consensus. During round one there will be an option for comments. These comments will be collated and may be included in round two of the survey.

Your responses to the rounds in this study will be kept confidential. However, your name and affiliation will be shared in the publication unless you indicate otherwise. You will be provided with an opportunity to choose to have your name and affiliation included in the demographics portion of the survey process.

**8. What will happen to information about me that is collected during the study?**

All the information collected from you for the study will be treated confidentially. The data will only be accessible to members of the research team. This data will be kept on a password protected server. The study results will be presented at conferences and in scientific publications. However, any observations and quotations in the material presented will be de-identified. The data will be securely retained, then destroyed 3 years after the project ends.

By providing your consent, you are agreeing to us collecting personal information about you for the purposes of this research study. Your information will only be used for the purposes outlined in this Participant Information Statement.

**9. What if I would like further information about the study?**

When you have read this information, Dr. Marian Reven, or any member of the ARQAT board will be available to answer any questions.

**10. When will this study begin and how long will it take?**

This study is set to begin on September 29, 2024, when an in-person and online meeting hosted by ARQAT is held at the Sonesta Airport Hotel in Nashville, TN, USA. During this 4-hour meeting, there will be an opportunity for the PI and the board of ARQAT to explain the study, answer questions, and launch the actual survey which will be sent to all participants approximately 2 weeks after this meeting. Everything is completed electronically and submitted via email. Attendance at this meeting is **not** necessary to participate in this study.

**There will be 4-6 weeks between Delphi rounds and the timeline is projected to be:**

| **Rounds** | **Emailed** | **Due back** | **Time & Task** |
| --- | --- | --- | --- |
| 1 | October 16, 2024 | November 6, 2024 | 3 weeks survey |
| Break for holidays |  |  | 8 weeks analysis |
| 2 | January 8, 2025 | January 29, 2025 | 3 weeks survey |
|  |  |  | 6 weeks analysis |
| 3 | March 12, 2025 | April 2, 2025 | 3 weeks survey |
|  |  |  | 2 weeks analysis |
| 4 (if needed) | April 16, 2025 | May 1, 2025 | 3 weeks survey |

**11. What if I have a problem or complaint about this study?**

This study is considered Non-Human Subject Research (NHSR) and is approved by the West Virginia University Institutional Review Board (IRB) #2205571104. Complaints or concerns can be directed to the PI, Dr. Marian Reven at [marian.reven@hsc.wvu.edu](mailto:marian.reven@hsc.wvu.edu).

For information regarding your rights as a participant in research or to talk about the research, contact the WVU Office of Human Research Protection (OHRP) at +1 (304) 293-7073 or by email at [IRB@mail.wvu.edu](mailto:IRB@mail.wvu.edu).

**12. Will I be told the results of this study?**

When analysis of the Delphi data is complete, researchers will provide participants with a summary of the findings. This will likely occur in late-2025.

**13. What do I do if I want to participate?**

Indicate your agreement to participate by using the link found in the Cover Letter. During the survey, you will be able to save your survey results and return to your survey as many times as you need during the 2 to 3-week survey period.

**References**

Harris, P. A., Taylor, R., Minor, B. L., Elliott, V., Fernandez, M., O’Neal, L., McLeod, L., Delacqua, G., Delacqua, F., Kirby, J., Duda, S. N., & REDCap Consortium. (2019). The REDCap consortium: Building an international community of software platform partners. *Journal of Biomedical Informatics*, *95*, 103208. https://doi.org/10.1016/j.jbi.2019.103208

Harris, P. A., Taylor, R., Thielke, R., Payne, J., Gonzales, N., & Conde, J. G. (2009). Research electronic data capture (REDCap) – A metadata-driven methodology and workflow process for providing translational research informatics support. *J Biomed Inform*, *42*(2), 377–381.
