# Supplementary material for: Essential criteria for reporting of aromatherapy-focused research in humans: An international Delphi consensus study protocol
Source: PLoS One. 2025 Mar 24;20(3):e0318379. doi: 10.1371/journal.pone.0318379 (PMC11932481; doi:10.1371/journal.pone.0318379)
Supplement: S2 File — (DOCX) [file pone.0318379.s002.docx]

Dear Prospective Participant,

This letter is a request for you to participate in a research project to produce a reporting guideline for aromatherapy research in humans. This project is being conducted by Dr. Marian Reven in the School of Nursing at WVU.

**Why:** We have identified a need for clarity. Please click on this link to the Participant Information Sheet learn more.

**What is required:** If you decide to participate, you will be asked to complete up to four rounds of an electronic Delphi survey process. Your participation in this project will take approximately one to three hours for each survey round. Each round will be a two week period between October 2024 and April 2025. You will receive email notification and reminder emails for follow up.

**Who can participate:** You are eligible to participate if you read and write in the English language and agree to complete all rounds of the Delphi process. Authors of published aromatherapy-focused research and their team members on the associated publication are invited.

**Optional:** A face to face meeting will take place on September 29, 2024, from 4 to 8pm. For the face to face meeting that takes place in Nashville, TN, US, no interpretation services will be provided. There will be online access to the meeting for those who wish to attend virtually. Those attending in person will receive a $100.00 USD travel stipend to participate in the face to face meeting.

**Confidentiality:** Your participation in this project will be kept as confidential as legally possible. Responses to the Delphi survey rounds will be deidentified before sending items into the next round. All data will be reported in the aggregate. Your participation is entirely voluntary. You may skip any question that you do not wish to answer, and you may discontinue at any time. The West Virginia University Institutional Review Board's review of this research project is on file with the WVU Office of Human Research Protections.

**Dissemination:** We intend to disseminate the results of this study in a high profile peer reviewed journal and will acknowledge all contributors.

If you have any questions about this research project, please feel free to contact me, the Principal Investigator, at +1 304-293-3399 or by email at [marian.reven@hsc.wvu.edu](mailto:marian.reven@hsc.wvu.edu). Additionally, you can contact the WVU Office of Human Research Protections at +1 304-293-7073.

I hope that you will participate in this research project, as it could help us better understand what is considered necessary to the complete and transparent reporting of aromatherapy-focused research in humans. Thank you for your time and consideration.

Sincerely,

Dr. Marian Reven

For Participant: I agree that I have read and understand what this project is about, and by clicking the link below, I agree to participate in the project.

If you have not already done so, please read the Participant Information Sheet, then follow the survey link to the Delphi Round 1 here:
