# Supplementary material for: Essential criteria for reporting of aromatherapy-focused research in humans: An international Delphi consensus study protocol
Source: PLoS One. 2025 Mar 24;20(3):e0318379. doi: 10.1371/journal.pone.0318379 (PMC11932481; doi:10.1371/journal.pone.0318379)
Supplement: S3 File — (DOCX) [file pone.0318379.s003.docx]

**
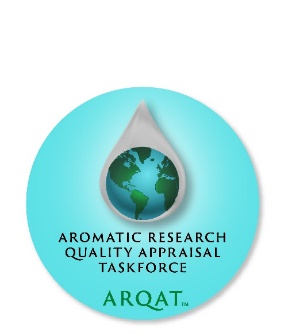
Explanatory document for “Items to be reported in aromatherapy research – Delphi consensus survey” hosted by the Aromatic Research Quality Appraisal Taskforce (ARQAT)***

***Adapted from TREATS and Explanatory Statement document (Reven et al., 2023).**

For further explanation and elaboration of ARQAT’s definitions of best practice in aromatherapeutic and essential oil research, visit [www.arqat.org](http://www.arqat.org) for the White paper, the TREATS, and Explanatory Statement document, and a list of resources.

**Section 1: Title**

See survey questions 1, 2, 3

**Section 2: Characterization of the essential oil(s)/ volatile extract(s)**

Q4 **Include the common and the botanical name of the essential oil(s)/volatile extract used for the intervention in the publication title.**

Essential oil (EO) name is provided including full binomial, also known as the botanical or scientific name, provided in Latin which provides identification of genus, species, and if applicable chemotype (if applicable – noted by ct or var). The binomial name may also include the attributed researcher.

Examples (not limited to):

Lavender: Lavandula angustifolia

Tea tree: Melaleuca alternifolia

Rosemary: Rosemary officinalis ct camphor

Rosemary: Rosemary officinalis var camphor

Bergamot (Citrus bergamia (Risso) (Wright & Arn.)

**Q5 Include the production method (steam distillation, carbon dioxide extraction, solvent extraction, cold-pressed extraction) of the essential oil/volatile extract.**

Extraction method of the EO is provided.

Examples (not limited to):

Steam distilled

Cold pressed or expressed

CO_2_ extraction

Solvent extraction (absolutes)

Fractional distillation

**Q6 Include information about the plant part used to create the essential oil/volatile extract.**

EO plant part extracted is provided.

Examples (not limited to):

- Flowers
- Leaves
- Root
- Bark
- Grasses
- Needles
- Rinds
- Resins

**Q7 Include the cultivation method that provides farming method details such as pesticide/herbicide use, organic methods, and how the plant was grown and harvested.**

Cultivation method provides farming method details such as:

- Pesticide/herbicide use
- Organic methods
- How the plant was grown and harvested (e.g., wildcrafted)

**Q8 Include the country of origin where the essential oil plant was grown and harvested.**

Where the EO plant material was grown and harvested is provided.

**Q9 Include the name of the company that manufactured and/or sold the essential oil/volatile extract.**

Manufacturer differs from distributer. Was the manufacturer of the EO clearly stated? A distributor will place their own marketing and label on the product. A supplier may be a warehouse that holds EOs on behalf of the distributor to sell, such as large marketplace companies.

Examples (false names used to create examples):

- John Doe’s Lavender Farm (manufacturer)
- The Essential Oil Warehouse (supplier)
- Happy Farm Aromatherapy Company (distributor)

**Q10 Include the batch number of the essential oil/volatile extract or identify that it was extracted in the researcher's lab.**

This is a unique number provided by the supplier that will allow the EO to be traced back through its journey from plant to bottle and provide authenticity to the EO.

**Q11 Include the identification of major or complete essential oil/volatile extract constituents listed as percentages and which analysis was performed, e.g. GC/FID, GC/MS, with Chiral Analysis, Headspace Analysis including analysis parameters, and which MS library was used for identification.**

Major or complete EO constituent percentages (chemical analysis) identified in study or as an attached link, such as gas chromatography, mass spectrometry (GC-MS) data.

##### Section 3: Rationale for study design and choice of plant materials

**Q12 The aromatherapy definition, rationale for the definition, and the source of the definition used for the intervention are provided.**

Consider if you would know how to replicate the intervention with the given description. Refer to Section 2 for what needs to be included. Aromatherapy research should also adhere to research standards including the following:

- Researchers adhered to stated protocols (Reliability)
- The interventionists and participants consistently adhered to the protocol that the researcher designed for the study (Fidelity)
- The protocols were not changed during the trial (Adds to fidelity and validity)

**The rationale for EO** selection is provided. Have the researchers addressed their rationale for using this intervention with this particular population: Age-appropriate, EO appropriate, blend appropriate?

**Q13 Describe the theoretical or conceptual framework as it relates to using aromatherapy in research in humans.**

While this is important in other qualitative research, we observe it is often not included in aromatherapy research. Examples of theoretical research frameworks are:

Whole system health

Comfort Theory (Katharine Kolcaba)

Chaos Theory (Margaret Wheatley)

**Q14 Describe the rationale for determining the selected essential oil(s) and application methods that are appropriate for the age and demographics of the participant population and that support the research study hypothesis.**

No further explanation offered

**Q15 The report contains a statement on whether the extract(s) used were obtained from species not classified as threatened or CITES-protected.**

See below

**Q16 For extracts sourced from threatened and/or CITES-protected species, the report contains the steps for the CITES exemption process, and a summary of the measures implemented to maintain ethical sourcing standards.**

***Sustainability and Clinical Aromatherapy/Volatile Extract Research***

***Background***

*In clinical research, there is a moral obligation to responsibly source plant extracts, particularly*

*from IUCN2 recognized threatened and near threatened species, and CITES3-protected species.*

*Doing so helps to safeguard biodiversity and the ecosystems where these fragile plants are*

*integral. This approach upholds our ethical duty to the environment and those who place their well-being in the hands of researchers. At least 18% of extracts used in aromatherapy worldwide are facing extinction (Airmid Institute,*

*2024). Ignoring core sustainability principles in the sourcing of extracts can exacerbate adulteration,*

*compromise replicability, and risk unethical sourcing by researchers and end-users.*

***Adulteration***

*Extracts linked to threatened, near threatened and CITES-protected plant species are commonly*

*adulterated due to their scarcity, exorbitant prices, cost barriers to securing permits, poor*

*processing, and inexperienced handling.*

***Safety***

*Adulterated extracts have safety implications. In clinical research, safety is paramount, and the use*

*of adulterated extracts raises significant concerns. Such extracts may introduce unknown*

*substances, increasing the risk of adverse reactions, including allergic responses and toxicity.*

*These impurities can result in unpredictable health effects, potentially compromising participant*

*well-being. Ensuring the use of pure extracts is essential for safeguarding against these risks and*

*upholding the safety standards of clinical studies.*

***Efficacy***

*Adulterated extracts have efficacy implications. In clinical research, the efficacy of treatments*

*using extracts is critically dependent on their purity. Adulterated extracts, diluted or modified*

*with additives, may result in diminished therapeutic effects, compromising the intended outcomes*

*of clinical trials. Such alterations can lead to unreliable data on the efficacy of extracts, which in*

*turn can affect the validity of the research. Therefore, the use of pure extracts is essential to*

*ensure accurate and reliable results in clinical studies.*

*1. Extracts collectively represent an essential oil, carrier oil, hydrolat, CO2 extract, and/or absolute.*

*2. IUCN: International Union for Conservation of Nature*

*3. CITES: Convention on International Trade in Endangered Species of Wild Fauna and Flora.*

***Chemistry***

*Understanding the isomeric makeup of a chemical constituent is vital during the procurement of extracts,*

*particularly of essential oils linked to species facing extinction. This level of detailed analysis can reveal*

*which part of the plant (e.g. heartwood or leaves) was utilized thereby guiding ethical sourcing decisions.*

***Replicability***

*To maintain research integrity and ensure sound replicability, it's vital to use confirmed pure extracts in*

*research. These extracts have standardized levels of active compounds, crucial for their bioactivity.*

*Variations in chemical profiles due to impurities can skew results and hinder validation of findings by*

*other researchers. Hence, purity in extracts is fundamental for credible research outcomes and*

*implications.*

***Unethical sourcing***

*Leading by example in protecting plants is crucial. Responsible sourcing of extracts used in*

*research showcases the impact and broader implications of using extracts from threatened, near*

*threatened, and CITES-protected species. It also raises questions about the application of*

*research findings in practice, and whether end-users will adhere to stringent and understood*

*ethical standards of sourcing.*

***Reference***

*Airmid Institute. (2024). Airmid Institute Biannual List of Threatened, Near Threatened, and*

*CITES-Protected Plants, Mammals, Fungi, and Seaweed Species Used in Aromatherapy,*

*Perfumery, and Aromatic Herbalism.* [*www.airmidinstitute.org*](http://www.airmidinstitute.org)

##### Section 4: Aromatherapist involvement and safe handling of essential oils

**Q17 Include a clear description of, and rationale for, the outcome measures used for aromatherapy-focused research.**

No further explanation provided.

**Q18 Identify that a qualified, registered, or certified aromatherapist is consulted in the design of the aromatic intervention.**

Identified that a qualified aromatherapist was consulted. There are several terms used to identify a qualified aromatherapist.

Examples (not limited to):

Registered aromatherapist

Certified aromatherapist

Qualified aromatherapist

Name of aromatherapy school or education provided

**Q19 Describe the aromatherapy expertise, background, and training of those providing the aromatic intervention.**

**Q20 Describe the safety considerations of the essential oil(s) relevant to the application method and dosage.**

**Safety considerations** while using the EO are acknowledged and provided.

Examples (not limited to):

Dilution for topical application

Participants asked about past allergic reactions or sensitivities to EOs or fragrances

Acknowledge if pregnant or nursing and include the rationale for exclusion (if excluded)

Identified EO safety measures specific to infants and children if they were included as study participants

Contraindications related to EOs and health condition/medications (e.g., asthma, seizures, hypertension)

Diffuser cleansing method discussed (if a diffuser was used in the study)

**Q21 Provide information of any allergic, idiosyncratic or adverse reactions to the essential oil or control, including actions taken, or acknowledge that no adverse reactions are reported.**

**Report of allergic and adverse reactions** to EO or control during the trial or comment that no adverse reactions are reported.

Examples (not limited to):

Report of participants with reactions

Types of reactions

Actions were taken to address reactions

**Q22 Describe how the essential oils are stored during the trial and how participants are instructed on storage.**

Examples (not limited to):

EO was protected from light and heat

EO storage location identified (e.g., refrigerator, locked cabinet)

Participants were instructed how to store EOs or trial materials

##### Section 5: Topical application methods and dosage regime

**Q23 Provide the dilution of the essential oil, including the volume or weight per volume, and the name of the diluent.**

**Dilution of EO** is provided by listing the volume or weight per volume and including the name of the diluent.

Examples (not limited to):

A 3% dilution of *Lavandula angustifolia* EO and cold-pressed sweet almond oil

Lavender was used at a dilution of 1ml per 100 ml almond oil (v/v)

**Q24 Provide the dose of the essential oil (amount given at one time).**

**Dose of EO** given to participants. Dose refers to the specified amount of essential oil given at one time.

Examples (not limited to):

5 mls lavender essential oil, 2% almond oil dilution

0.25ml dose of the 1% EO infused cream

**Q25 Describe the body part and surface area to which the essential oil is applied (e.g. hands, 10 cm).**

Examples (not limited to):

10 ml of a 2% v/v blend of lavender EO in sweet almond oil was applied to the entire surface of the back, neck, and shoulders

2 ml of a 1% v/v blend of rose EO in jojoba oil was applied to the forehead and temples of the face

**Q26 Provide the frequency of the essential oil dose.**

**The frequency of the EO** dose is provided. How often? Once a day, twice a day, every so many hours? (Dosage regimen is frequency plus duration)

**Q27 Describe the duration of the intervention in days, weeks, or months.**

**The duration of the EO treatment** is described. How many days, weeks, or months? How long were participants studied? (Dosage regimen is frequency plus duration)

**Q28 Describe the control or placebo used in the intervention.**

**Description of the control or placebo** is provided.

Examples (not limited to):

Usual treatment (no control intervention)

Carrier oil (jojoba, almond oil, etc.)

Another essential oil

A specific fragrance

An “attention control” (non-equivalent alternative activity)

**Q29 Patient adherence with dosage regime is reported (where relevant).**

Reporting patient adherence to the protocol is crucial, as otherwise the results are flawed.

**Q30 Provide the diluent common and binomial name.**

**Diluent or carrier(s) including the name and binomial (Latin)** is provided. If a diffuser is used, the type of water is provided.

Examples (not limited to):

Almond oil: *Prunus amygdalus var dulcis*

Jojoba oil: *Simmondsia chinensis*

**Q31 Provide the source of the diluent or carrier of the delivery system.**

**Source of carrier or delivery system.** Manufacturer differs from distributer. Was the manufacturer of the carrier oil clearly stated? A distributor will place their own marketing and label on the product. A supplier may be a warehouse that holds carrier oils on behalf of the distributor to sell, such as large marketplace companies. Examples (false names used to create examples):

John Doe’s Almond Oil Factory (manufacturer)

The Essential Oil Warehouse (supplier)

Happy Farm Aromatherapy Company (distributor)

##### Section 6: Inhalation methods and dosage regime

**Q32 Describe the mode of aromatic inhalation and the delivery device used.**

**Mode of inhalation** is provided.

Direct (personal inhaler, aroma stick, sniff stick, cotton ball, patch)

Indirect (ambient, diffusion)

**Q33 Provide the total dose of the essential oil(s)/volatile oil(s), including the approximate distance the device is from the nose e.g. micro liters per inhalation.**

**Dose of EO.** Total Dosage of EO given to participants is provided. Total volume is given: how many mls of EO? The approximate distance of the inhaler device from the nose is described.

Examples (not limited to):

0.25 ml *Lavandula angustifolia* placed in a personal inhaler stick and held 3 inches from the nares for 5 seconds (**Best option** due to dose, distance, and duration listed)

0.5 ml *Lavandula angustifolia* placed on 2x2 felt pad attached to shirt collar (**less useful option** due to measurement difficult to replicate)

2 drops of *Lavandula angustifolia* were placed on the cotton ball; the patient was instructed to gently move back and forth approximately 6 inches from the nose (**less useful option** due to drops listed)

**Q34 Provide the frequency of the essential oil dose.**

**Frequency of EO.** The frequency of the dose is provided. How often? Once a day, twice a day, every so many hours? (Dosage regimen is frequency plus duration)

**Q35 Provide the duration of the intervention.**

No additional information provided.

**Q36 Describe the placebo or control used in the intervention including the aroma (if any) and volume used.**

**Description of control or placebo** is provided.

Examples (not limited to):

Usual treatment (no control intervention)

Carrier oil (jojoba, almond oil, etc.)

Another essential oil

A specific fragrance

Distilled water

An “attention control” (non-equivalent alternative activity)

**Q37 Provide the diluent common and binomial name (if applicable), and state what odor it has, if any.**

**Carrier(s) including the name, binomial (Latin),** or the volume of distilled water both measured in ml. While carrier oils are not necessary for inhalation purposes, they are used to dilute the essential oil in some situations.

Examples (not limited to):

Almond oil: *Prunus amygdalus var dulcis*

Jojoba oil: *Simmondsia chinensis*

120 mls distilled water placed in a diffuser

**Q38 State the suppliers of the diluent, carrier, and delivery system.**

No additional information provided.

##### Section 7: Participant olfactory capacity and experience

**Q39 Participants are asked if they are experiencing anosmia (loss of smell), parosmia, hyposmia or other olfactory disorders.**

**Anosmia** is addressed. Were participants asked if they currently experience loss of smell? Anosmia is not necessarily a reason for exclusion; however, consideration is important.

**Q40 Participants are asked about any previous use of essential oils**

Participants were asked about their previous use of essential oils and their preference or aversion to certain essential oils. This provides valuable information on how to proceed. It also gives researchers insight into EO preferences. The Discovery of an aversion to the EO(s) being used in the trial may exclude participants from study enrollment.

**Q41 Participants are asked about their preferences or aversions to any essential oils.**

No additional information provided.

**Q42 Test environment odor control is described.**

No additional information provided.

**Q43 Prior to the intervention, participants are asked about their expectations of the essential oil(s) used in the intervention. If the odor is recognized, it is acknowledged in the publication**

**Participants’ expectations** were addressed. Participants were asked how they thought the aroma of the EO would affect them, particularly if they recognized it (e.g., “I expected Lavender to be relaxing”). If the odor was recognized, this was acknowledged under limitations.

**Q44 Prior to the intervention, participants are asked about their odor preference and like or dislike of the essential oil(s) aroma used in the intervention. Any biases are acknowledged under the limitations section.**

**Odor preference bias** was addressed. Participants were asked whether they liked or disliked the aroma of the treatment and control aromas. Biases were acknowledged under limitations.

**Q45 Participants are asked about odor recognition related to the essential oil(s) used in the intervention. If the odor is recognized, it is acknowledged in the publication.**

No additional information provided.

**Q46 Participants are asked about their perceived aroma intensity of the essential oil(s) used in the intervention, and variations described. If the aroma is perceived as too weak or too strong, it is acknowledged in the publication.**

**Perceived aroma intensity** was addressed. Examples (not limited to):

Participants were asked if they found the aroma too strong or too weak

Researchers addressed that the treatment and control aromas were perceived to be similar strengths (if applicable)

Olfactory fatigue was addressed (experience of losing sensitivity to odors after prolonged exposure)

**Q47 Discuss olfactory fatigue (experience losing sensitivity to odors after prolonged exposure, habituation) if applicable to the study.**

**Q48 Adverse effects from olfaction testing are reported as "none" or with a description of adverse effects.**

Participants’ responses were recorded to address adverse effects. Examples (not limited to):

Irritating or non-irritating responses such as sneezing, watery eyes, excess salivation (potential trigeminal nerve response), headache, and nausea.

**Reference**

Reven, M. E., Bowles, E. J., Audia, D. D., Cohen, M. M., Joswiak, D. J., Kurkas Lee, B. A., May-Fitzgerald, A. C., Peppers-Citizen, M., Resnick, J. A., Tomaino, J. M., & Unger, B. J. (2023). Quality Appraisal of Research Reporting for Aromatherapy and Essential Oil Studies in Humans: Proposed Checklist for “Transparent Reporting for Essential oil and Aroma Therapeutic Studies.” *Journal of Integrative and Complementary Medicine*. https://doi.org/10.1089/jicm.2023.0006
