# Supplementary material for: Essential criteria for reporting of aromatherapy-focused research in humans: An international Delphi consensus study protocol
Source: PLoS One. 2025 Mar 24;20(3):e0318379. doi: 10.1371/journal.pone.0318379 (PMC11932481; doi:10.1371/journal.pone.0318379)
Supplement: S4 File — (DOCX) [file pone.0318379.s004.docx]

S4. Aromatic Research Quality Appraisal Taskforce (ARQAT) past and present members

<https://www.arqat.org/what-is-arqat>

Board

Dr. Marian Reven (President)

Dr. E. Joy Bowles (Vice-President)

Dr. Marilyn Peppers-Citizen (Treasurer)

Ms. Amanda May-Fitzgerald (Secretary)

Dr. Kelly Ablard

Ms. Denise Joswiak

Ms. Bethany Unger

Dr. Jerelyn Resnick

Ms. Michele Cohen

Advisors

Ms. Donna Audia

Dr. Janet Tomaino

Ms. Barb Kurkas Lee

Mr. William McGilvray
