# Supplementary material for: Essential criteria for reporting of aromatherapy-focused research in humans: An international Delphi consensus study protocol
Source: PLoS One. 2025 Mar 24;20(3):e0318379. doi: 10.1371/journal.pone.0318379 (PMC11932481; doi:10.1371/journal.pone.0318379)
Supplement: Attachment — Submitted filename: Request for revisions dated 100424.docx [file pone.0318379.s005.docx]

**Request for revisions dated 10/4/24**

**PONE-D-24-41981**

**Essential criteria for reporting of aromatherapy-focused research in humans: An international Delphi consensus study protocol**

**Recruitment wording changes to manuscript**

Line 132 Original “Recruitment draws from the global community aiming to include as many countries as possible.”

Lines132-134 Updated “Recruitment began in August 5, 2024, and will end when the first survey in the series is sent out, estimated to be October 16, 2024. Recruitment draws from the global community aiming to include as many participants from as many countries as possible.”

Lines 257-258 Original “At the time of manuscript submission, the research will have just commenced recruitment of participants.”

263-264 Update “At the time of manuscript submission, the research is recruiting participants from 8/5/24 through 10/16/24.

**IRB oversight updates**

Lines 254-255 Original “Ethical approval has been granted from the WVU Institutional Review Board (IRB) #2205571104.”

Lines 255-261 Updated “This study was submitted for ethical approval to the WVU Institutional Review Board (IRB) #2205571104. Per the WVU IRB, this study qualifies as Non-Human Subjects Research (NHSR). As such, full consent is not required. However, the WVU IRB requires that all activities involving collecting/using information about humans include a question asking for their voluntary permission and an explanation of the activity.  The following standard text is included in the Cover letter, “I voluntarily agree to participate and I understand that I can stop participating at any time (Yes/No).”
